# Security Analysis of a Federated Learning Framework for Medical Image-to-Image Translation

**DOI:** 10.1007/s10916-026-02436-8

**Published:** 2026-07-04

**Authors:** Ciro Benito Raggio, Lina Bucher, Oliver Blanck, Francesco Cicone, Paolo Zaffino, Maria Francesca Spadea

**Affiliations:** 1 Institute of Biomedical Engineering, Karlsruhe Institute of Technology, Fritz-Haber-Weg 1, 76131 Karlsruhe, Baden-Württemberg Germany; 2 Department of Physics and Astronomy, Heidelberg University, Im Neuenheimer Feld 226, 69120 Heidelberg, Baden-Württemberg Germany; 3 Department of Radiation Oncology, University Hospital Schleswig-Holstein, Feldstrasse 21, 24105 Kiel, Schleswig-Holstein Germany; 4 Department of Experimental and Clinical Medicine, Magna Graecia University, Viale Europa, 88100 Catanzaro, Calabria Italy

**Keywords:** Federated learning, Image-to-image translation, Synthetic computed tomography, Security attacks

## Abstract

Federated Learning (FL) emerged as a privacy-preserving paradigm for collaborative training of deep learning models across institutions without sharing patient data. This approach has been applied to complex tasks such as medical image-to-image (I2I) translation, including MRI-to-synthetic CT (sCT) generation. However, existing federated I2I frameworks often assume privacy preservation as an inherent property of FL rather than a requirement to be explicitly validated, leaving their robustness to representative adversarial threat scenarios largely unexplored. In this study, we evaluated the vulnerability of a federated MRI-to-sCT translation framework (FedSynthCT-Brain) to three representative attack classes: Deep Leakage from Gradients (DLG), Federated Membership Inference Attack (FedMIA), and data poisoning. The efficacy of corresponding defense mechanisms, such as Secure Aggregation (SecAgg) and Byzantine-robust median aggregation (FedMedian), were assessed. DLG enabled only the recovery of coarse anatomical structures, with no clinically identifiable details (SSIM $\le$ 0.16, PSNR $\le$ 11 dB) across clients, suggesting limited vulnerability under the evaluated DLG setting. In contrast, FedMIA achieved high membership discrimination, with AUC scores between 0.92 and 0.99, revealing a critical privacy vulnerability. The introduction of SecAgg reduced AUC values to near-random levels (0.23–0.56) across all centers without impacting synthesis quality. Under high-noise poisoning, the standard federated averaging (FedAvg) aggregation rendered the federation inoperative, while FedMedian restored performance close to the no-poisoning baseline in most scenarios, with significant residual degradation in specific center configurations. At low noise levels, the advantage of FedMedian was less consistent, as low-level noise injection may be indistinguishable from natural heterogeneity across centers, potentially enabling stealthy degradation. These findings demonstrate that federated I2I translation frameworks are not inherently secure and require explicit, multi-layered evaluation. As FL is increasingly adopted in clinical workflows, our results underscore the necessity of integrating cryptographic, algorithmic, and infrastructural safeguards for secure deployment.

## Introduction

Deep Learning (DL) transformed medical imaging by enabling models to learn complex mappings between modalities directly from data [1]. Within this landscape, image-to-image (I2I) translation emerged as a prominent framework, encompassing tasks such as inter-sequence MRI translation and MRI- and Cone-Beam Computed Tomography (CBCT)-to-CT synthesis [2, 3]. Among these, synthetic CT (sCT) generation has attracted particular clinical interest in radiotherapy treatment planning, as it eliminates the need for dedicated CT acquisition, thus removing associated ionizing radiation dose and co-registration errors [3].

However, developing effective DL models requires large, diverse, multi-center datasets [4, 5], which institutions often cannot share due to regulatory constraints such as the GDPR [6, 7] and patient privacy requirements. This motivated the adoption of Federated Learning (FL), which enables collaborative training by exchanging only model updates rather than raw patient data [8]. Beyond its application in classification and segmentation tasks, FL has been successfully applied to federated MRI cross-modality translation [9–11]. More recently, it has been extended to sCT synthesis through FedSynthCT-Brain [12], a framework for brain MRI-to-sCT translation across four centers, and further extended to CBCT-to-sCT translation [13].

These approaches demonstrated effective cross-center generalization without requiring the exchange of patient data. However, none of these frameworks included an explicit security evaluation, treating privacy preservation as an inherent property of FL rather than a design requirement to be verified. Although FL mitigates direct data sharing, federated systems remain vulnerable to well-established adversarial threats, including privacy leakage from shared updates and integrity attacks that can compromise model behavior [14–16]. Prior work on FL security in medical imaging focused primarily on augmentation, classification and segmentation tasks [17], such as backdoor attacks in GAN-based data augmentation [18], poisoning for classification [19], and evasion attacks via adversarial perturbations in classification tasks [20].

To the best of the authors’ knowledge, no study has evaluated security threats in the context of federated I2I translation. This work investigated representative adversarial threats and defense strategies, analyzing their trade-off between clinical utility, computational feasibility, and robustness.

## Related Work

The security of FL systems encompasses two principal threat dimensions, including privacy attacks, which target the extraction of sensitive information from model updates, and integrity attacks, which aim to corrupt the global model through malicious updates. Both classes of attacks exploit the exchange of model updates in the FL protocol, which constitutes a primary attack surface in the absence of dedicated security mechanisms [15, 16].

Within privacy-related attacks, membership inference attacks (MIAs) exploit the tendency of overfitted models to behave differently on training versus unseen data. Nasr et al. [21] showed that FL models are not inherently more resistant to membership inference than centralized counterparts, and that client update vectors extend the attack surface. Zhu et al. proposed FedMIA [22], exploiting aggregated update information across clients and rounds. In addition, prior studies have investigated client participation inference from update dynamics, highlighting that even coarse-grained aggregation may still expose sensitive information about client involvement [23, 24].

In contrast to privacy-oriented attacks, integrity threats, such as data poisoning and backdoor attacks, represent a growing trend. Bagdasaryan et al. [25] demonstrated that a single compromised client can inject a targeted backdoor via model replacement within a single aggregation round, with subsequent work exploring increasingly stealthy strategies [26, 27]. In medical imaging, poisoning has been studied for classification [19] and federated GAN-based data augmentation [18], where the downstream impact is ultimately measured on classification performance. The implications for I2I translation are fundamentally different, as compromised synthesis models may introduce subtle intensity distortions directly propagated into clinical workflows (i.e., radiotherapy dose calculation), where such errors are difficult to detect using standard evaluation metrics.

Beyond MIAs and poisoning, gradient-based attacks represent another major privacy leakage vector. Zhu et al. demonstrated that private training data can be reconstructed from shared gradients via iterative optimization (Deep Leakage from Gradients), achieving pixel-wise accuracy on image data [28]. Subsequent work showed that reconstruction quality degrades with larger batch sizes [29], and batch normalization statistics substantially hinder standard attacks [30]. Furthermore, recent work extended inversion to diffusion-based reconstruction in medical imaging [31].

Several complementary defense strategies have been proposed [15, 16]. For instance, differential privacy (DP) [32] adds calibrated noise to client updates, providing formal privacy guarantees against gradient inversion and membership inference [21, 29], at the cost of a utility trade-off particularly concerning in medical image synthesis. Secure aggregation (SecAgg) [33] prevents individual gradient exposure via cryptographic commitments, but offers no protection against poisoning. Byzantine-robust aggregation [34] mitigates integrity threats by down-weighting anomalous updates, but provides no privacy guarantees.

Despite extensive research on FL security, prior studies primarily focused on classification, segmentation, and data augmentation tasks. Consequently, established privacy and integrity attacks, as well as corresponding defense mechanisms, have not been evaluated in I2I translation settings, particularly in clinically relevant synthesis tasks such as sCT generation. To address this gap, we implemented three well-established attack vectors against FedSynthCT-Brain [12]: (i) deep leakage from gradients (DLG), (ii) federated membership inference, and (iii) data poisoning. Furthermore, SecAgg and FedMedian were implemented as representative defense strategies.

## Materials and Methods

### Datasets

The study included 102 patients across five centers (A–E). Centers A–C represented institutional datasets from the US, Italy, and Germany, while Centers D and E were extracted from the SynthRAD Grand Challenge 2023 [35]. Detailed dataset characteristics (i.e., scanner specifications, acquisition protocols) were reported in Table 1. Following the original FedSynthCT-Brain [12] implementation, each training center (Centers A, B, C, D) partitioned data into training, validation, and test sets to maximize training cases. Overall, this corresponded to a 70/10/20 split. Center E was used strictly for the external evaluation and generalization assessment of the federated model and thus never participated in model training.

Notable heterogeneity was observed across sites regarding scanner vendors, field strengths, and voxel spacing. The MRI acquisitions included both 1.5T and 3T systems, while the CT scans were acquired with varying tube voltages (120–140 kVp) and heterogeneous reconstruction settings. The dimensions of the original images also varied considerably across centers, particularly for Centers C, D and E.

**Table 1:** Overview of dataset characteristics across the five participating centers. The table reports patient counts, image dimensions, scanner models, acquisition parameters, and voxel spacing for both MRI and CT modalities, highlighting inter-center heterogeneity

		Centers				
		A	B	C	D	E
	Patients	15	14	21	29	23
	Original dimension	$256\times 176\times 256$	$256\times 176\times 248$	226−357 $\times$512 $\times$ 512	167− 213 $\times$216−262 $\times$250−277	167− 262 $\times$200−225 $\times$225−248
MRI	Voxel size [mm$^3$]	$1\times 1\times 1$	$1\times 1\times 1$	$0.78\times 0.78\times 1$	0.98 −1.12$\times$ 0.98 −1.12$\times$0.98 −1.12	$0.98\times 0.98\times 0.98$
	Scanner	MAGNETOM Trio	Biographm MR	Vantage Titan	MAGNETOM Avanto_fit,Skyra, Vida_fit,Prisma_fit	MAGNETOM Aera,Avanto_fit
	Field strength [T]	3	3	1.5	1.5–3	1.5–3
CT	Voxel size [mm$^3$]	0.49−0.67 $\times$ 0.49−0.67 $\times$2.5	0.98$\times$0.98$\times$3.27	0.78$\times$0.78$\times$1	0.69−0.78 $\times$0.69−0.79 $\times$1−3	0.59−1.27 $\times$0.59−1.27 $\times$1−2
	Scanner	LightSpeed QX/i	Discovery ST	Brilliance Sensation Open	Brilliance Big Bore	SOMATOM Definition AS
	Tube voltage [kVp]	140	120	120	120	120

The pre-processing was performed at each client level, including rigid registration of MRI and CT volumes. N4 bias correction [36] and normalization to [0,1] were applied to MRI volumes. Subsequently, all MRI and CT volumes were resampled to a fixed dimension of $256\times 256\times 256$ using a combination of cropping, resizing, and constant padding. Non-anatomical regions (i.e., scanner couch and background) were masked, assigning -1000 HU to CT and 0 to MRI [12].

### Federated Learning Framework

The FL workflow was implemented using the Flower framework [37], while model development and training were performed in PyTorch [38] and MONAI [39]. Following the original implementation, the model aggregation was performed via FedAvg, with local updates regularized using the FedProx [40] strategy, which introduces a proximal term in the local objective to limit the deviation from the global model. The proximal coefficient was set to $\mu =3$ [12].

Furthermore, as delineated in the original implementation, a 2D UNet-based model [41] (with $\approx 2.68\times 10^7$ trainable parameters) was employed as the foundation for all experiments, thereby facilitating the examination of the attacks on the original framework. The network consisted of four encoding levels with channel depths of 64, 128, 256, and 512, followed by a bottleneck block that doubled the channel count to 1024 prior to upsampling. Downsampling was performed via max pooling, and upsampling employed transposed convolutions with a kernel size of 4 and a stride of 2. Batch normalization was applied after each convolutional layer. LeakyReLU activations were used in the encoder and standard ReLU activations in the decoder. Skip connections then concatenated the encoder feature maps to the corresponding decoder levels [41]. Data augmentation was applied during training. Specifically, random spatial transformations were used, including left-right flipping along the spatial axis, random rotations, and random translations, implemented using MONAI.

Local training was performed using the Random Multi-2D approach for 1 epoch per round [12]. Optimization was carried out using Adam with a fixed learning rate of $1 \times 10^{-4}$ and a batch size of 8 slices.

The federated training was conducted for 20 communication rounds. Client participation followed a full participation strategy, where all available clients (Centers A–D) contributed in each round.

Model selection and evaluation were performed by selecting the final global model obtained at the last communication round.

### Experimental Scenarios

**Fig. 1:**
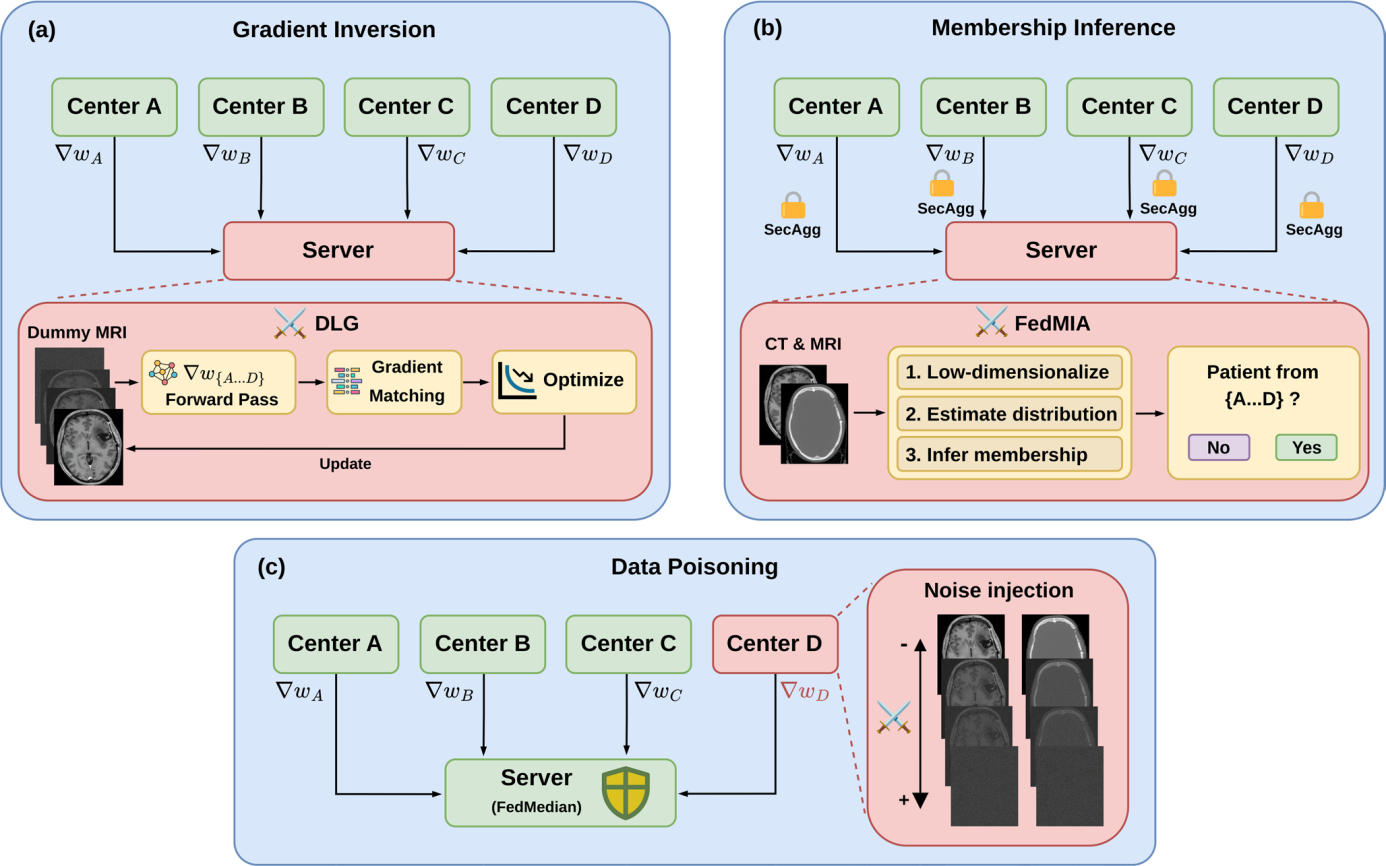
Overview of the three attack scenarios and corresponding defenses evaluated in the proposed federated sCT synthesis framework. ***(a) Gradient Inversion:*** a honest-but-curious server attempts to reconstruct private MRI training data from the gradients $\nabla w_{\{A\ldots D\}}$ via the DLG attack, which iteratively optimizes a dummy input through gradient matching. ***(b) Membership Inference:*** the FedMIA attack exploits client gradients to determine whether a given CT–MRI pair was included in the training set of any participating center; SecAgg is adopted as a countermeasure, encrypting each client’s gradient prior to server-side aggregation. ***(c) Data Poisoning:*** a malicious client corrupts the federation by injecting additive noise into its local training data, thereby altering the contributed gradient; FedMedian aggregation is employed at the server as a robust defense against such poisoned updates

Three categories of attacks were simulated within the federated framework, as illustrated in Fig. 1: (a) gradient inversion, (b) membership inference, and (c) data poisoning. Where applicable, corresponding defense mechanisms were evaluated within the same experimental setting.

#### Gradient Inversion

The DLG attack [28] was implemented with the aim of reconstructing local inputs from shared gradients. Therefore, in this scenario, the sensitive data to reconstruct corresponded to the input MRIs. For each attack, a 2D dummy MRI slice was initialized as a learnable tensor within the anatomical mask. The reconstruction was performed by minimizing the gradient matching loss, defined as the squared difference between dummy and real gradients, computed exclusively within the anatomical mask to suppress background reconstruction.

The Adam and the Limited-memory Broyden-Fletcher-Goldfarb-Shanno (L-BFGS) optimizers [28] were evaluated separately. For Adam, a learning rate of $l_r = 0.1$ was used with a StepLR scheduler reducing $l_r$ by a factor of $\lambda = 0.1$ every 10, 000 iterations. For L-BFGS, a line search step size of 0.1 was adopted. Both optimizers were run for 30, 000 iterations.

In this scenario, no explicit defense mechanism was required, and the analysis was conducted under the baseline [12] federated setting, while limiting the batch size to 1 to minimize DLG optimization complexity.

#### Membership Inference

For this scenario, the FedMIA [22] strategy was implemented. The attack targeted the server, which had access to the global model checkpoints at each round and to the client model updates uploaded prior to aggregation. The objective was to determine, for a given data sample, whether it was part of the training set of a specific target client. According to the FedMIA implementation, the attack followed a three-step procedure:

1. For each communication round *r*, the client update was defined as:
  1 $$\begin{aligned} \Delta w_k^{(r)} = w_k^{(r)} - w^{(r)} \end{aligned}$$
  where $w_k^{(r)}$ represents the local model of client *k* and $w^{(r)}$ the global model. The cosine similarity between the gradient of the loss, computed on the global model with respect to each candidate sample (one MRI and CT batch), and the flattened $\Delta w_k^{(r)}$ was subsequently calculated, forming an $n_{\text {clients}} \times n_{\text {batches}}$ similarity matrix.
2. The non-membership distribution was estimated by fitting a Gaussian distribution $\mathcal {N}(\mu , \sigma ^2)$ to the similarity scores of non-target clients, where $\mu$ and $\sigma$ represent the mean and standard deviation, respectively, and excluding samples with scores greater than $\mu + 3\sigma$ associated with high similarity and consequently potential membership.
3. The membership probability was computed as the Gaussian cumulative distribution function (CDF) evaluated at the target client score, with higher values indicating greater membership likelihood.

In order to establish the membership, in the simulated scenario the attacker had access to the original MRI and CT data, providing an upper bound on attack and, consequently, defense effectiveness.

To mitigate this threat SecAgg [33] was applied. Under this protocol, individual client updates $\Delta w_k^{(r)}$ are masked through cryptographic pairwise secrets prior to transmission, ensuring that the server can only access the aggregated sum of updates without being able to reconstruct any individual contribution. This mechanism directly affects the assumptions underlying the FedMIA attack, which requires access to per-client update vectors $\Delta w_k^{(r)}$ to compute cosine similarity scores against candidate samples. Therefore, the application of SecAgg precludes the observation of these vectors, thereby rendering the calculation of similarity and, consequently, the estimation of membership probabilities, no longer viable. The experiments employed the built-in SecAgg+ [42] implementation provided by the Flower framework, which follows the additive masking scheme proposed by Bonawitz et al.

The FedMIA attack was subsequently re-evaluated under this aggregation setting to assess whether the attack pipeline remains applicable and to quantify the resulting privacy risk mitigation.

#### Data Poisoning

The impact of the data poisoning attack was assessed by corrupting the training data of a client during the federated training process. The primary objective of the attack was to degrade the performance of the global model on the remaining clients by injecting corrupted data into the federation. The poisoning strategy was implemented by introducing additive Gaussian noise to the MRI and CT volumes. For each patient, noise *n* was sampled from a Gaussian distribution with mean $\mu _x$ and standard deviation $\sigma _x$ computed individually from the original CT or MRI volume *x*, and blended with it according to a noise level parameter $\gamma \in [0, 1]$, where $\gamma = 0$ preserves the original image and $\gamma = 1$ replaces it entirely with noise, according to:

2 $$\begin{aligned} x_{\text {poisoned}} = (1 - \gamma ) \cdot x + \gamma \cdot n, \quad n \sim \mathcal {N}(\mu _x, \sigma _x) \end{aligned}$$

The value of $\gamma$ varied across three noise levels $\gamma \in \{0.1, 0.5, 1.0\}$. These correspond to 10%, 50% and 100% noise injection, respectively.

Subsequently, in order to quantify both vulnerability and robustness, the impact of data poisoning was evaluated under different aggregation strategies. As a baseline configuration, the standard FedAvg aggregation combined with FedProx [12] was first considered in the absence of adversarial attacks, establishing an upper bound on synthesis performance. The poisoning attack was then applied under the FedAvg aggregation combined with FedProx to measure the degradation induced by a compromised client for each client in the federation (Centers A, B, C, D).

As a potential defense mechanism, a Byzantine-robust aggregation strategy based on coordinate-wise median (FedMedian) [43] was evaluated as an alternative to FedAvg. In contrast to mean-based aggregation, FedMedian mitigates the influence of anomalous updates by selecting the median value across client parameters for each coordinate, thereby limiting the impact of outliers introduced by poisoned data. The integration with FedProx was maintained to ensure the closest possible alignment with the original framework.

### Evaluation Metrics

Image synthesis quality and similarity were quantified using complementary metrics capturing both pixel-wise accuracy and perceptual fidelity [3, 12].

The Mean Absolute Error (MAE) measured pixel-wise prediction error between the synthesized image $I_{\text {Synth}}$ and the ground-truth image $I_{\text {GT}}$ in Hounsfield units (HU), and is defined as:

3 $$\begin{aligned} \textrm{MAE} = \frac{1}{N} \sum _{i=1}^{N} \left| I_{\text {Synth}}^{(i)} - I_{\text {GT}}^{(i)} \right| , \end{aligned}$$

where *N* is the total number of voxels.

The Peak Signal-to-Noise Ratio (PSNR) evaluated reconstruction the fidelity by comparing the maximum possible signal intensity $I_{\max }$ to the Mean Squared Error (MSE), defined as:

4 $$\begin{aligned} \textrm{PSNR} = 10 \log _{10} \left( \frac{I_{\max }^2}{\textrm{MSE}} \right) , \end{aligned}$$

where

5 $$\begin{aligned} \textrm{MSE} = \frac{1}{N} \sum _{i=1}^{N} \left( I_{\text {syn}}^{(i)} - I_{\text {gt}}^{(i)} \right) ^2. \end{aligned}$$

The Structural Similarity Index (SSIM) assessed perceptual similarity by jointly comparing luminance, contrast, and structural information between image patches and was defined as:

6 $$\begin{aligned} \textrm{SSIM}(x,y) = \frac{(2\mu _x \mu _y + C_1)(2\sigma _{xy} + C_2)}{(\mu _x^2 + \mu _y^2 + C_1)(\sigma _x^2 + \sigma _y^2 + C_2)}, \end{aligned}$$

where $\mu _x$ and $\mu _y$ denoted the mean intensities, $\sigma _x^2$ and $\sigma _y^2$ the variances, and $\sigma _{xy}$ the covariance of image patches *x* and *y*. Constants $C_1$ and $C_2$ were used to stabilize the division.

In the context of membership inference attacks, model privacy leakage was evaluated using the Area Under the Receiver Operating Characteristic Curve (AUC-ROC). The ROC curve represents the trade-off between the True Positive Rate (TPR) and False Positive Rate (FPR), defined as:

7 $$\begin{aligned} \textrm{TPR} = \frac{\textrm{TP}}{\textrm{TP} + \textrm{FN}}, \quad \textrm{FPR} = \frac{\textrm{FP}}{\textrm{FP} + \textrm{TN}}, \end{aligned}$$

where TP, FP, TN, and FN denote true positives, false positives, true negatives, and false negatives, respectively. Consequently, the AUC summarized the ROC curve into a single scalar value, with higher values indicating stronger attack performance and thus greater privacy risk [22].

## Results and Discussion

**Fig. 2:**
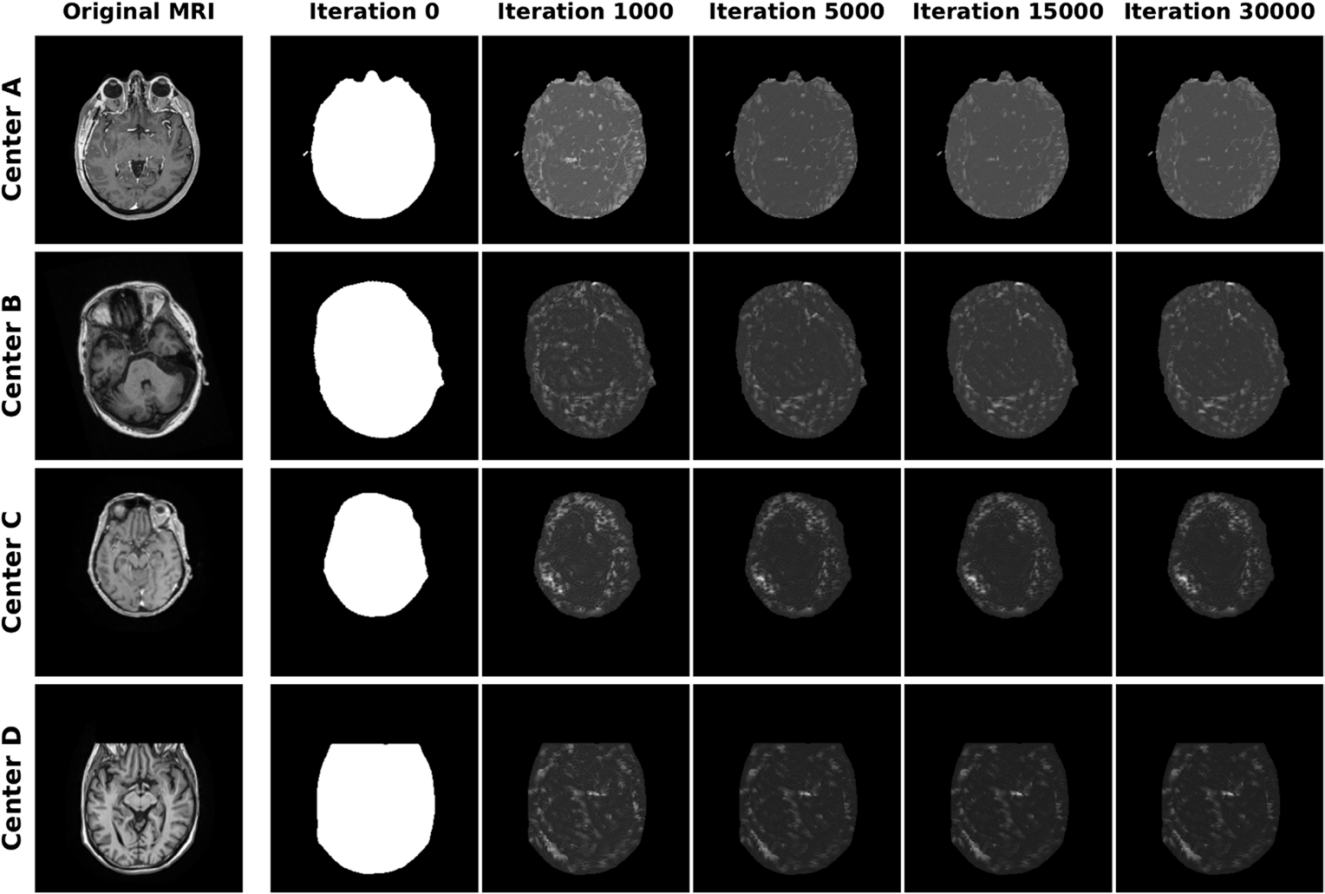
DLG-based reconstruction across federated centers. Reconstructions evolved from a uniform initialization to coarse anatomical shapes within the first 5, 000 iterations, with no further qualitative improvement beyond 15, 000 iterations

As described in Section “Gradient Inversion”, the DLG attack was performed using both the Adam and L-BFGS optimizers over 30, 000 iterations to ensure stable convergence. L-BFGS exhibited a slower and less stable behavior, with the gradient matching loss reaching a plateau after $\approx 10{,}000$ iterations. In contrast, Adam provided more stable optimization. However, as demonstrated in Fig. 2, the reconstruction improved within the initial 5, 000 iterations from a uniform initialization toward the anatomical shape of the original MRI, with no qualitative improvement beyond 15, 000 iterations. The reconstructed images failed to preserve identifiable anatomical details despite capturing coarse structural shapes, with SSIM of $0.16\pm 0.05$ and PSNR of $11\pm 2$ dB at optimizer convergence for the reference cases presented in Fig. 2, demonstrating the limited vulnerability of the framework against this attack. This can be attributed to multiple factors, including architectural and training factors, as well as the federated paradigm.

The UNet architecture employed in this study (proposed in [12]) incorporates batch normalization layers by design, which have been shown to substantially hinder gradient inversion attacks [30]. Furthermore, the federated training context implies that gradients are computed over multi-patient batches rather than single samples, a condition known to degrade reconstruction quality in gradient inversion settings [29]. The implemented DLG configuration represented an upper bound assessment of gradient inversion risk. The adoption of a reduced batch size resulted in an increased rate of information leakage per sample, thereby providing a more favorable scenario for the attacker in comparison to realistic FL implementations. In practical scenarios where gradients are aggregated across multiple samples per batch, the reduced granularity of updates is expected to further compromise the DLG’s reconstruction performance.

**Fig. 3:**
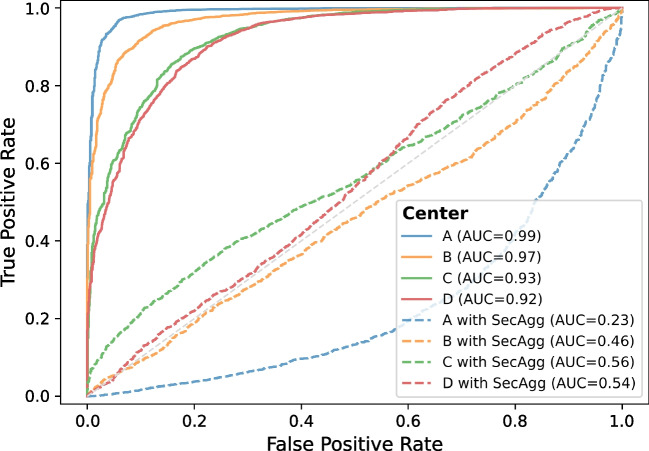
ROC curves for membership inference attack (FedMIA) across clients, before and after the implementation of SecAgg, evaluated on MRI and CT batches at the final training round. In the original framework, the attack achieved high discrimination at all centers, indicating severe vulnerability to membership inference. The application of SecAgg resulted in a substantial decrease in AUC values, thereby indicating the efficacy of SecAgg in mitigating the attack

The FedMIA attack was evaluated across all four training centers (A-D). As shown in Fig. 3, using the original aggregation strategy (FedAvg+FedProx) [12], the attack achieved accurate discrimination for all centers, with AUC values between 0.92 and 0.99 on the received model and client updates of the final training round. These results confirm that, within the original framework [12], the server could reliably identify whether a specific patient sample might have been used in the training of a particular client, thereby highlighting a substantial privacy vulnerability.

The implementation of SecAgg substantially reduced the attack efficacy, as evidenced by the AUC values, which decreased to 0.23, 0.46, 0.56, and 0.54 for Centers A, B, C, and D, respectively (see Fig. 3). Notably, this reduction held even under a best-case attacker setting, where access to the original MRI and CT data provided an upper bound on attack effectiveness. Furthermore, the impact of SecAgg on model performance, as presented in Table 2, was found to be negligible. The MAE, SSIM, and PSNR values exhibited consistency across all centers before and after the implementation of SecAgg, with variations occurring within the reported results of the original framework baseline [12]. Overall, these results indicate that SecAgg provides robust privacy protection against FedMIA without compromising synthesis performance.

As detailed in Section “Data Poisoning”, the data poisoning attack was simulated by corrupting the training data of one client at a time (Centers A–D) at three different noise levels ($\gamma \in \{0.1, 0.5, 1.0\}$). The impact was then evaluated on all clients and on the external Center E. The results obtained under all scenarios are reported in Table 3 in terms of MAE, considered the most sensitive similarity metric in this context [12].

**Table 2:** Model performance across centers before and after applying Secure Aggregation (SecAgg) as a defense mechanism against Federated Membership Inference Attacks (FedMIA). Results are reported as mean and standard deviation

Center	SecAgg	MAE (HU)	SSIM	PSNR (dB)
A	X	$92.9\pm 2.8$	$0.96\pm 0.01$	$33.4\pm 0.6$
	$\checkmark$	$87.0\pm 2.1$	$0.96\pm 0.01$	$33.7\pm 0.5$
B	X	$122.9\pm 6.3$	$0.84\pm 0.02$	$21.2\pm 1.2$
	$\checkmark$	$121.2\pm 6.6$	$0.84\pm 0.02$	$21.3\pm 1.3$
C	X	$96.0\pm 6.3$	$0.81\pm 0.03$	$21.6\pm 1.4$
	$\checkmark$	$94.3\pm 9.5$	$0.81\pm 0.03$	$21.6\pm 1.4$
D	X	$68.5\pm 12.0$	$0.92\pm 0.01$	$26.5\pm 1.0$
	$\checkmark$	$68.9\pm 10.1$	$0.92\pm 0.01$	$26.5\pm 1.0$
E	X	$102.4\pm 12.5$	$0.88\pm 0.03$	$26.1\pm 1.7$
	$\checkmark$	$100.6\pm 10.6$	$0.88\pm 0.03$	$26.1\pm 1.7$

**Table 3:** Quantitative evaluation of the impact of data poisoning attacks on federated sCT synthesis. MAE [HU] is reported for each center under three noise levels ($\gamma \in \{0.1, 0.5, 1.0\}$) – indicating 10%, 50% and 100% of noise injected respectively – comparing FedAvg (top) and FedMedian (bottom) aggregation strategies. The no-poisoning baseline is reported in the last row. Bold values indicate the better-performing aggregation strategy for each center–noise

**FedAvg**						
		Evaluated Centers – MAE [HU]				
Poisoned Center	Noise	A	B	C	D	E (External)
A	100%	–	$208.2 \pm 22.2$	$215.4 \pm 21.8$	$210.6 \pm 38.6$	$192.3 \pm 16.9$
	50%	–	$140.4 \pm 13.3$	$106.5 \pm 16.6$	$92.7 \pm 17.7$	$121.6 \pm 12.8$
	10%	–	$124.2 \pm 7.1$	$\mathbf {94.3 \pm 13.3}$	$\mathbf {70.5 \pm 9.1}$	$110.9 \pm 11.1$
B	100%	$183.3 \pm 11.1$	–	$236.0 \pm 24.1$	$248.5 \pm 25.7$	$222.8 \pm 14.5$
	50%	$92.8 \pm 5.5$	–	$99.9 \pm 18.8$	$84.0 \pm 16.5$	$117.3 \pm 12.4$
	10%	$92.1 \pm 1.8$	–	$\mathbf {93.9 \pm 14.6}$	$\mathbf {72.9 \pm 12.3}$	$\mathbf {103.5 \pm 9.9}$
C	100%	$504.4 \pm 28.3$	$841.6 \pm 38.1$	–	$682.8 \pm 57.3$	$623.7 \pm 50.8$
	50%	$138.8 \pm 15.0$	$165.0 \pm 17.4$	–	$143.7 \pm 26.7$	$180.4 \pm 18.1$
	10%	$95.3 \pm 2.4$	$131.5 \pm 9.6$	–	$79.1 \pm 12.2$	$119.0 \pm 11.5$
D	100%	$764.9 \pm 36.9$	$790.0 \pm 36.8$	$740.3 \pm 20.7$	–	$787.0 \pm 26.0$
	50%	$186.3 \pm 19.9$	$228.7 \pm 25.7$	$181.9 \pm 24.2$	–	$207.0 \pm 19.3$
	10%	$94.9 \pm 1.7$	$134.5 \pm 8.0$	$98.8 \pm 11.7$	–	$120.5 \pm 11.1$
No poisoning	–	$\mathbf {87.0 \pm 1.7}$	$118.8 \pm 7.9$	$\mathbf {95.0 \pm 9.1}$	$\mathbf {69.4 \pm 8.3}$	$\mathbf {107.1 \pm 11.2}$
**FedMedian**						
		Evaluated Centers – MAE [HU]				
Poisoned Center	Noise	A	B	C	D	E (External)
A	100%	–	$\mathbf {124.1 \pm 12.6}$	$\mathbf {104.1 \pm 9.4}$	$\mathbf {91.5 \pm 12.2}$	$\mathbf {115.2 \pm 11.1}$
	50%	–	$\mathbf {117.1 \pm 9.7}$	$\mathbf {97.3 \pm 16.9}$	$\mathbf {80.7 \pm 12.4}$	$\mathbf {115.5 \pm 12.1}$
	10%	–	$\mathbf {115.3 \pm 7.5}$	$97.1 \pm 11.4$	$75.6 \pm 10.5$	$\mathbf {110.6 \pm 11.5}$
B	100%	$\mathbf {92.9 \pm 3.6}$	–	$\mathbf {105.2 \pm 12.2}$	$\mathbf {82.2 \pm 10.0}$	$\mathbf {111.4 \pm 10.0}$
	50%	$\mathbf {87.6 \pm 0.8}$	–	$\mathbf {95.4 \pm 12.7}$	$\mathbf {72.0 \pm 8.2}$	$\mathbf {114.7 \pm 11.7}$
	10%	$\mathbf {88.3 \pm 2.6}$	–	$96.3 \pm 11.9$	$74.7 \pm 9.0$	$108.2 \pm 10.5$
C	100%	$\mathbf {88.7 \pm 9.8}$	$\mathbf {118.4 \pm 14.6}$	–	$\mathbf {99.0 \pm 12.0}$	$\mathbf {124.3 \pm 11.0}$
	50%	$\mathbf {83.9 \pm 1.5}$	$\mathbf {109.8 \pm 10.5}$	–	$\mathbf {83.8 \pm 14.6}$	$\mathbf {119.3 \pm 12.8}$
	10%	$\mathbf {85.3 \pm 0.2}$	$\mathbf {110.4 \pm 6.9}$	–	$\mathbf {75.1 \pm 9.9}$	$\mathbf {108.9 \pm 11.3}$
D	100%	$\mathbf {85.0 \pm 4.1}$	$\mathbf {122.5 \pm 14.1}$	$\mathbf {126.6 \pm 21.0}$	–	$\mathbf {130.6 \pm 10.4}$
	50%	$\mathbf {83.1 \pm 0.9}$	$\mathbf {109.6 \pm 8.6}$	$\mathbf {96.3 \pm 13.3}$	–	$\mathbf {113.6 \pm 12.0}$
	10%	$\mathbf {86.9 \pm 1.8}$	$\mathbf {113.3 \pm 10.1}$	$\mathbf {94.7 \pm 16.3}$	–	$\mathbf {108.5 \pm 10.2}$
No poisoning	–	$89.2 \pm 4.9$	$\mathbf {108.6 \pm 5.3}$	$98.1 \pm 10.1$	$74.1 \pm 9.5$	$108.6 \pm 11.2$

**Fig. 4:**
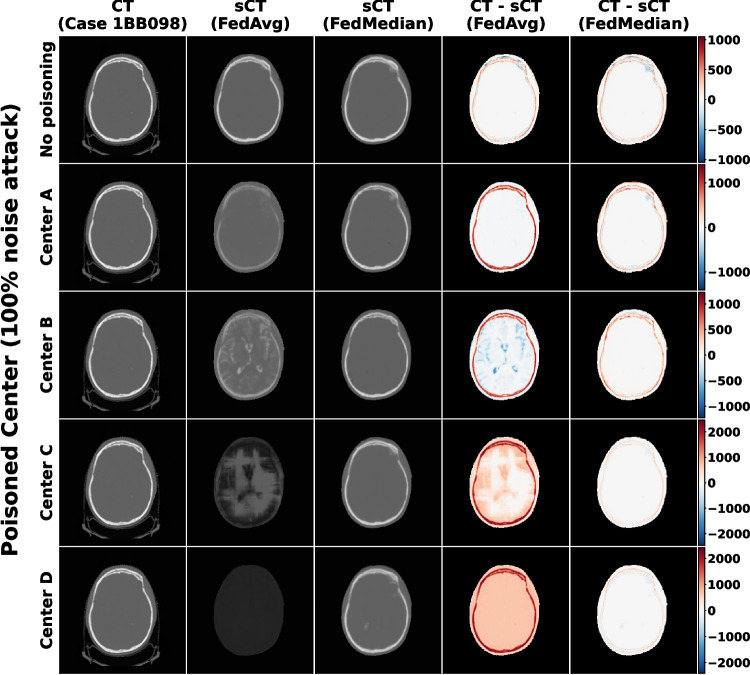
Visual comparison of sCT for a representative patient from external Center E under a 100% noise data poisoning attack ($\gamma = 1.0$), corresponding to the worst-case scenario. The first row represents the no-poisoning baseline, while the other rows correspond to a different poisoned training center (Centers A-D). Columns show, from left to right, the reference CT, the sCT generated with FedAvg and FedMedian aggregation, and the corresponding pixel-wise difference maps (CT − sCT). FedMedian consistently restored image quality across all poisoning configurations, while FedAvg produced severely corrupted and invalid outputs

Under FedAvg, the application of high-noise poisoning ($\gamma = 1.0$ – 100%) led to severe degradation of the model across all non-poisoned centers, thereby impeding the effective operability of the federation. Notably, the most severe degradation was observed when Center C was poisoned, where FedAvg yielded MAE values of 504 HU, 841 HU, and 682 HU for Centers A, B, and D, respectively. These outcomes effectively render the model technically and clinically inoperative (see Fig. 4). A similar impact was observed when Center D was poisoned at the same noise level, with MAE values exceeding 740 HU across all non-poisoned centers. This greater impact can be attributed to the larger amount of data provided by Center C (21 patients) and Center D (29 patients) [12]. At intermediate noise levels ($\gamma = 0.5$ – 50%), performance degradation remained critical, with MAE reaching high values (i.e., 229 HU) when Center D was poisoned. In conditions of low noise ($\gamma = 0.1$ – 10%) poisoning, the operability of the federation was preserved with MAE values tending to the no-poisoning baseline. However, more substantial degradations were observed in specific center combinations, thereby confirming the impact of data poisoning also at low noise levels. The FedMedian aggregation strategy consistently mitigated or severely limited the attack under all poisoning scenarios at $\gamma = 1.0$ and $\gamma = 0.5$, often reducing MAE to the no-poisoning baseline across all center pairs (see Table 3). For instance, under a 100% noise injection on Center D, FedMedian achieved a substantial MAE reduction ($>80$%) across all centers compared with FedAvg. However, a residual degradation persisted on Center C, where the MAE ($\approx 127$ HU) remained about 30 HU higher than the no-poisoning baseline, suggesting a potentially non-negligible impact in clinical settings. At 10% noise injection, the advantage of FedMedian over FedAvg was less consistent, and in several configurations FedAvg achieved equivalent MAE. This finding suggests that a $10\%$ noise injection may not be sufficiently distinguishable from the natural heterogeneity of the data distribution across centers, which can originate from differences in scanner resolution or intrinsic acquisition noise. Notably, this observation aligns with a poisoning attack strategy, under which malicious clients inject deliberately low levels of noise to evade detection while still cumulatively degrading the global model performance. In a clinical context, particularly with regard to radiotherapy dose calculation workflows, these subtle and systematic distortions may potentially propagate directly into the sCT-based dose computation pipeline. Such distortions may not be captured by routine quality assurance checks that primarily assess gross anatomical plausibility rather than voxel-wise HU accuracy. In contrast to tasks where model corruption results in immediately observable categorical errors, sCT synthesis models may instead manifest spatially coherent but quantitatively biased HU estimates. This observation highlights a notable limitation of aggregation strategies that are oriented towards robustness. While such strategies may prove effective in mitigating high-intensity attacks, they can remain partially vulnerable to low-amplitude, stealthy poisoning strategies.

Notably, the external Center E followed the same trend as the clients, demonstrating that the impact of poisoning extended beyond the federated training clients and that FedMedian effectively limited it in the external evaluation setting as well. Nevertheless, under FedMedian aggregation, the external Center E remained substantially affected under the 100% and 50% scenario, with MAE values ranging from 111 HU to 130 HU depending on the poisoned client, representing a consistent degradation with respect to the no-poisoning baseline of 108 HU. Indeed, statistically significant degradation (p-value $<0.05$) was observed in most of these cases, with the exception of Center B poisoned at 100% (p-value $>0.05$). Furthermore, a qualitative evaluation of the impact of data poisoning is presented in Fig. 4, showing the central–axial sCT slice of a representative patient from the external Center E under the worst-case attack scenario ($\gamma = 1.0$, i.e., 100% noise injection). This enables a direct comparison across poisoned training centers (Centers A–D) and the no-poisoning baseline. In detail, while poisoning Center A or Center B resulted in a degraded output, poisoning Centers C or D led to complete loss of structural information under FedAvg. In contrast, FedMedian demonstrated a consistent recovery of sCT in all poisoning scenarios, although some artifacts were observed (see FedMedian sCT - Poisoned Center D in Fig. 4).

However, the Differential Privacy defense mechanism was not investigated in this study. Although DP provides formal probabilistic privacy guarantees [32], its noise-injection mechanism introduces a performance-privacy trade-off [44] that poses a particular challenge for sCT synthesis, where voxel-level HU accuracy directly conditions downstream dosimetric calculations. Consequently, SecAgg was identified as the optimal solution for defending against the FedMIA threat model at the communication level. Nevertheless, additional investigation of DP for federated sCT synthesis may be a viable direction for future research.

## Conclusion

This study evaluated the vulnerability of a federated MRI-to-sCT translation framework to three representative adversarial attack classes –gradient inversion, membership inference, and data poisoning– and assessed the efficacy of corresponding defense mechanisms. The DLG attack recovered only coarse anatomical structure without clinically identifiable detail, suggesting that the combination of architectural choices and federated training characteristics, together with the complexity of the I2I task, may constitute a substantial barrier to pixel-accurate gradient inversion.

Nevertheless, the gradient inversion was performed in 2D, at the same granularity as the information exposed during training and aggregation. A comprehensive 3D formulation of gradient inversion under 3D (or patch-3D) training settings could represent a noteworthy direction for future investigations of privacy leakage in volumetric federated medical imaging workflows.

The FedMIA attack demonstrated near-perfect membership discrimination in the baseline framework, confirming that federated I2I translation models are not inherently secure. However, the application of SecAgg reduced AUC values to random levels across all centers without any substantial impact on synthesis quality. Data poisoning under original settings (FedAvg and FedProx) [12] caused severe synthesis degradation at high noise levels, while FedMedian consistently restored model performance close to the no-poisoning baseline, at the cost of some artifacts observed in the sCTs of the external Center E. Collectively, these findings demonstrate that federated I2I translation frameworks, as well as other FL applications, require explicit security evaluation. The application of well-established strategies (such as SecAgg and FedMedian) represent viable, complementary defenses against privacy and integrity threats. However, results also demonstrated that low levels of noise (i.e., 10%) injected by a malicious client could be difficult to detect and mitigate. Although Byzantine-robust aggregation methods, such as Krum [45], represent a promising candidate for future investigation, their practical implementation necessitates a minimum number of participating clients that exceeds the federation size examined in this study. Future work should therefore explore detection mechanisms compatible with small-scale federations, such as cosine similarity-based gradient screening [46]. Furthermore, although no measurable computational or communication overhead was observed for SecAgg at the federation scale considered in this study, the cost of cryptographic secure aggregation protocols is known to scale with the number of participating clients [33, 42]. Therefore, future studies involving larger federations should explicitly profile these overheads.

In light of these findings, the adoption of encryption protocols and additional infrastructure-level security measures (i.e., strong authentication protocols) appears to be a necessary complement to algorithmic defenses. While FL is regarded as a privacy-preserving and secure solution, its resilience is contingent upon the absence of dedicated countermeasures. As evidenced by the FedMIA results, the absence of SecAgg in a baseline federated framework renders FL critically susceptible to membership inference attacks under the right conditions. This highlights the necessity of addressing security in FL at multiple levels, including algorithmic, cryptographic, and infrastructural aspects. Further studies should directly incorporate a security analysis when evaluating federated frameworks for medical I2I translation. When also combined with clinical studies, this would allow for a comprehensive assessment of the clinical implications of both attacks and defenses. Notably, this study was conducted with a limited number of clients. While this configuration is consistent with prior FL studies in the field of I2I translation, its application to large-scale clinical deployments is not fully reflected. Therefore, the observed security behavior in this work should be interpreted in the context of a small-scale federation. Future work should investigate larger federations with varying client numbers and controlled heterogeneity levels to better characterize the stability of attack and defense mechanisms under large-scale deployment conditions.

## Data Availability

Restrictions apply to the availability of the data supporting the findings of this study from Centers A, B and C which were used under licence for this study and are therefore not publicly available. The data from Center D and Center E were extracted from the public SynthRAD2023 Grand Challenge dataset and are available at 10.5281/zenodo.7260705.
